# Mobile Phone–Based Interventions for Smoking Cessation Among Young People: Systematic Review and Meta-Analysis

**DOI:** 10.2196/48253

**Published:** 2023-09-12

**Authors:** Xinmei Zhou, Xiaowen Wei, Anqi Cheng, Zhao Liu, Zheng Su, Jinxuan Li, Rui Qin, Liang Zhao, Ying Xie, Zhenxiao Huang, Xin Xia, Yi Liu, Qingqing Song, Dan Xiao, Chen Wang

**Affiliations:** 1 National Center for Respiratory Medicine; State Key Laboratory of Respiratory Health and Multimorbidity; National Clinical Research Center for Respiratory Diseases; Institute of Respiratory Medicine, Chinese Academy of Medical Sciences; Department of Tobacco Control and Prevention of Respiratory Diseases, Center of Respiratory Medicine, China-Japan Friendship Hospital, Beijing, China; 2 China-Japan Friendship School of Clinical Medicine, Capital Medical University, Beijing, China; 3 Graduate School, Chinese Academy of Medical Sciences and Peking Union Medical College, Beijing, China; 4 School of Health Policy and Management, Chinese Academy of Medical Sciences & Peking Union Medical College, Beijing, China; 5 School of Population Medicine and Public Health, Chinese Academy of Medical Sciences & Peking Union Medical College, Beijing, China; 6 Chinese Academy of Medical Sciences and Peking Union Medical College, Beijing, China; 7 National Center for Respiratory Medicine; State Key Laboratory of Respiratory Health and Multimorbidity; National Clinical Research Center for Respiratory Diseases; Institute of Respiratory Medicine, Chinese Academy of Medical Sciences, Beijing, China

**Keywords:** smoking cessation, young people, mobile health, text messaging, mHealth, PRISMA

## Abstract

**Background:**

Mobile phone–based cessation interventions have emerged as a promising alternative for smoking cessation, while evidence of the efficacy of mobile phone–based smoking cessation programs among young people is mixed.

**Objective:**

This study aimed to determine the efficacy of mobile phone–based interventions compared to usual practice or assessment-only controls on smoking cessation in young people.

**Methods:**

In this systematic review and meta-analysis, we searched Cochrane Library, Embase, PubMed, and Web of Science on March 8, 2023. We included randomized controlled trials that examined the efficacy of mobile phone–based interventions on smoking cessation in young people (age ≤30 years). The risk of bias was assessed with Cochrane Risk of Bias 2.

**Results:**

A total of 13 eligible studies, comprising 27,240 participants, were included in this analysis. The age range of the participants was between 16 and 30 years. Nine studies were SMS text messaging interventions, and 4 studies were app-based interventions. The duration of the smoking cessation intervention varied from 5 days to 6 months. The included studies were conducted in the following countries: the United States, China, Sweden, Canada, Switzerland, and Thailand. The meta-analysis revealed that SMS text messaging interventions significantly improved continuous abstinence rates compared to inactive control conditions (risk ratio [RR] 1.51, 95% CI 1.24-1.84). The subgroup analysis showed pooled RRs of 1.90 (95% CI 1.29-2.81), 1.64 (95% CI 1.23-2.18), and 1.35 (95% CI 1.04-1.76) for continuous abstinence at the 1-, 3-, and 6- month follow-up, respectively. Pooling across 7 studies, SMS text messaging interventions showed efficacy in promoting 7-day point prevalence abstinence (PPA), with an RR of 1.83 (95% CI 1.34-2.48). The subgroup analysis demonstrated a significant impact at the 1- and 3-month follow-ups, with pooled RRs of 1.72 (95% CI 1.13-2.63) and 2.54 (95% CI 2.05-3.14), respectively, compared to inactive control conditions. However, at the 6-month follow-up, the efficacy of SMS text messaging interventions in promoting 7-day PPA was not statistically significant (RR 1.45, 95% CI 0.92-2.28). In contrast, app-based interventions did not show significant efficacy in promoting continuous abstinence or 7-day PPA. However, it is important to note that the evidence for app-based interventions was limited.

**Conclusions:**

SMS text messaging–based smoking cessation interventions compared to inactive controls were associated with abstinence among young people and could be considered a viable option for smoking cessation in this population. More research is needed on smoking cessation apps, especially apps that target young people. Future research should focus on identifying the most effective mobile phone–based cessation approaches and on developing strategies to increase their uptake and intention.

## Introduction

### Background

Tobacco use is one of the leading risk factors for premature morbidity and mortality worldwide [1]. Smoking among young people is of particular concern. Despite the well-documented health risks associated with tobacco use, many young people continue to smoke or experiment with smoking. The prevalence of smoking among young people is especially troubling, as this age group is in the midst of crucial physical and psychological development. The harmful effects of smoking at this stage of life can have lifelong consequences, including increased risk of chronic disease, impaired cognitive function [2], and reduced quality of life [3]. In 2019, an estimated 155 million (95% uncertainty interval 150-160) people aged 15-24 years worldwide were tobacco smokers, with a prevalence of 20.1% in males and 4.95% in females [4]. Quitting before the age of 30 years can prevent more than 97% of the excess mortality caused by continued smoking [5]. Given the serious health risks associated with smoking, quitting is critical for young people.

While traditional cessation methods such as pharmacotherapy [6] and behavioral counseling [7] can be effective, their widespread implementation at a population level faces barriers [8]. Mobile phone–based smoking cessation interventions have emerged as a promising alternative to assist with smoking cessation [9-12]. Phone interventions are a cost-effective use of health care resources [13]. These interventions can provide personalized interactive support that is tailored to individual needs and characteristics, irrespective of location and time constraints [14,15], making them a valuable approach for smoking cessation in this demographic. Furthermore, young individuals are more open to novel and innovative approaches [16]. According to the International Telecommunications Union, approximately 66% of the global population had internet access in 2022 [17]. While previous research has suggested that SMS text message–based smoking cessation interventions were more effective than minimal smoking cessation support in the general population [18], evidence of the efficacy in young people remained inconclusive [19,20]. As countries work toward achieving the goal of reducing the prevalence of tobacco use [21], timely data on the efficacy of mobile phone–based smoking cessation programs among young people are necessary to guide effective policy and planning.

### Objective

To the best of our knowledge, there are no meta-analyses supporting the efficacy of mobile phone–based smoking cessation interventions among young people. The aim of this meta-analysis was to determine the efficacy of mobile phone–based smoking cessation interventions, excluding pharmacological treatment, in helping young smokers to quit.

## Methods

We adhered to the PRISMA (Preferred Reporting Items for Systematic Reviews and Meta-Analyses) guidelines for systematic reviews of interventions. We used a prespecified protocol registered with PROSPERO (CRD42022318845).

### Search Strategy and Selection Criteria

We included randomized controlled trials (RCTs) with young smokers (30 years or younger) who wanted to quit. Included trials had to be clearly focused on smoking cessation using SMS text messaging or a smoking cessation app without pharmacotherapy, compared to a control intervention. Trials that had a focus on pregnant women were not eligible for inclusion.

### Data Extraction

Studies were assessed for inclusion if they reported cigarette smoking cessation as the primary outcome. Self-reported abstinence from cigarette smoking and biochemically validated measures of abstinence were used to define smoking cessation.

Data extracted from each study included the study location, study design, population, inclusion criteria, exclusion criteria, follow-up period, details of the intervention group, details of the control group, definition of smoking cessation, number of participants, and smoking cessation rates. Wherever possible, an intention-to-treat analysis was used.

The following electronic bibliographic databases were last searched in March 2023: Cochrane Tobacco Addiction Group’s Specialised Register (Source: PubMed, Embase, ClinicalTrials.gov, and the ICTRP), Embase, PubMed, and Web of Science. The search strategies used in the Cochrane Library, PubMed, Embase, and Web of Science are listed in Multimedia Appendix 1. The database literature search was restricted to the English language and studies on humans. The search terms were text messaging, phone-based, smartphone, app, mobile health, sms, txt, young, student, adolescent, and smoking cessation. Both abstracts and full manuscripts were considered.

### Statistical Analysis

Authors XZ, XW, and AC independently confirmed study eligibility. Authors JL, YX, ZH, XX, YL, QS, and XZ extracted data, which were then checked by a second author (RQ, LZ, or AC). Two authors (XZ, AC, ZL, or ZS) independently assessed quality using the Cochrane Risk of Bias tool. All differences were resolved by discussion.

We used random-effects meta-analysis to analyze pooled outcome data among smokers who used SMS text messaging or an app compared with a control. Binary outcomes were estimated using risk ratios (RRs) and 95% CIs, with priority given to intention-to-treat data when available. For smoking cessation, meta-analyses were conducted for continuous abstinence and 7-day point prevalence abstinence (PPA). For 2 studies [22,23], data specifically for individuals 30 years and younger were extracted from the original data set and reanalyzed. The 7-day PPA at the 1-month follow-up for one study was derived from the third figure in Chulasai et al [24]. Heterogeneity between studies was assessed using the *I*^2^ statistic. A subgroup analysis of the length of follow-up was also performed. Additionally, we performed a sensitivity analysis by removing the studies with a high risk of bias. All analyses were performed using R 4.2.0 (R Foundation for Statistical Computing) and Revman 5.4 (The Cochrane Collaboration). We did not perform funnel plot asymmetry because no outcome had more than 10 studies in the meta-analysis [25].

The risk of bias for studies was assessed using the Cochrane Risk of Bias 2 tool [26]. Studies were considered to be at high risk in the domain of missing outcome data if the overall loss to follow-up was more than 50% or if there was a difference in follow-up rates of more than 20% between study arms.

## Results

**Figure 1.**
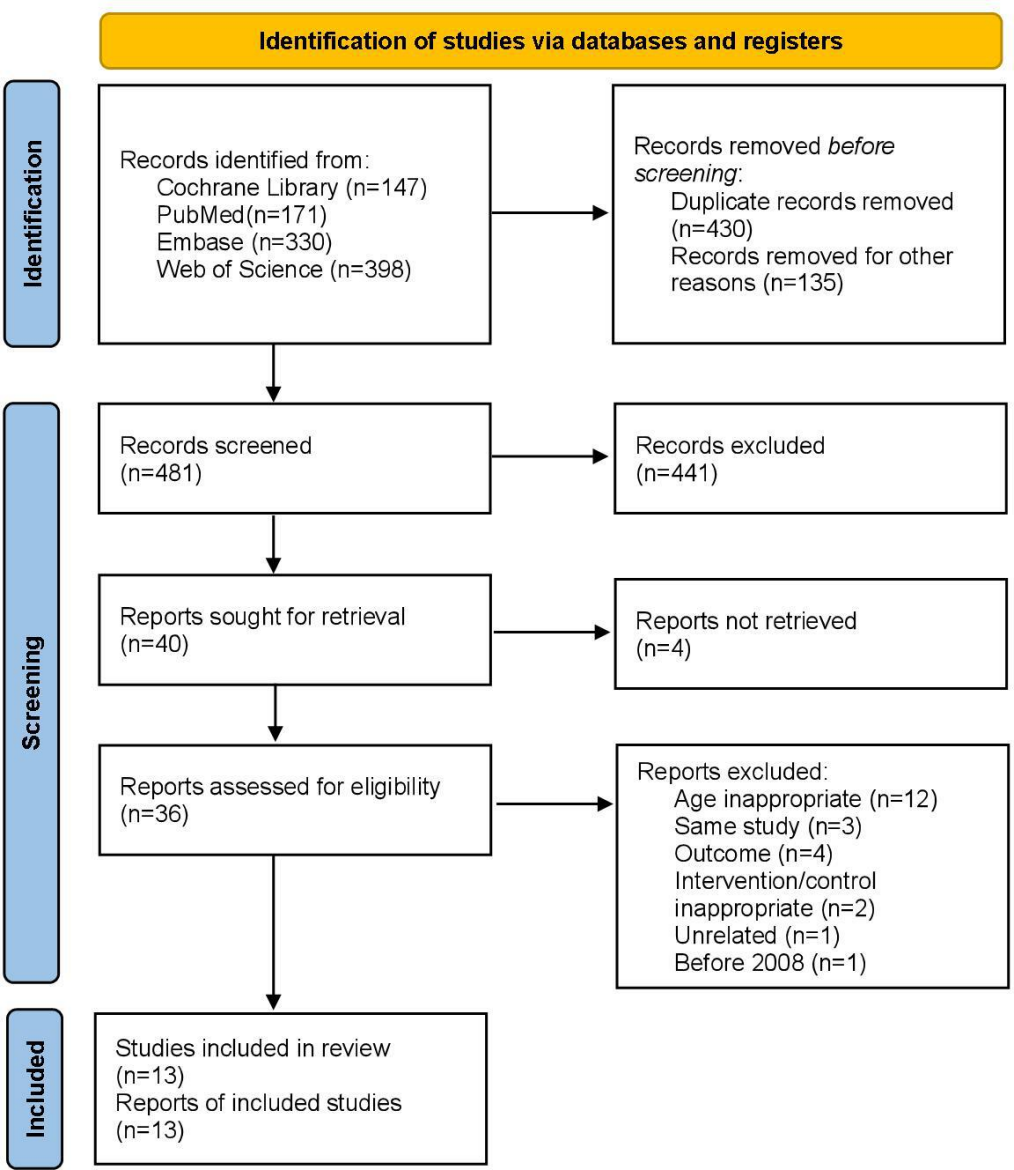
PRISMA (Preferred Reporting Items for Systematic Reviews and Meta-Analyses) flow diagram of evidence search and selection.

We identified 1046 full-text trial reports or titles and abstracts (Figure 1) and identified 13 RCTs [10,15,19,20,22-24,27-33] for inclusion in the final review. The complete process is shown in Figure 1.

### General Characteristics of the Selected Studies

Details of the eligible studies are presented in Table 1. A total of 13 eligible studies, comprising 27,240 participants, were included in this analysis. The age range of the participants was mainly between 16 and 30 years. Of the 13 included studies, 2 were cluster RCTs [29,32] and 11 were individual RCTs. There were 5 studies in the United States [15,28,31,33,34], 2 studies in Sweden [10,20], 2 studies in China [22,32], and 1 study each in Canada [19], Switzerland [29], and Thailand [24]. One study was conducted online [23], in which registered users of the Smoke Free app were the study participants, regardless of location. Measures of current smoking varied between studies: 4 studies [10,20,29,32] included daily smokers and weekly smokers, 2 studies [19,22] included only daily smokers, and Palmer et al [31] used a definition related to daily smoking (vaping nicotine at least 25 days/month). Three studies [15,24,28] included participants who smoked monthly or more, and Ybarra et al [34] included participants who smoked 24 cigarettes or more per week (at least 4 per day on at least 6 days in a week). One study used the definition of having smoked 100 cigarettes in life and now smoking every day or some days [33]. The remaining study [23] did not report the definition of smoker. Of the 13 studies, 5 recruited from vocational schools [29,32], high schools [20], or colleges [10,24]. The remaining studies recruited smokers from the community, health care facilities, online, or a combination of sources.

**Table 1.** Characteristics of included studies.

Study	Participants, n	Age (years)	Intervention	Control	Outcome
Baskerville et al [19], 2018, Canada, RCT^a^	1599	19-29	Crush the Crave Application	On the Road to Quitting–Self Help: The control group received an evidence-based self-help guide known as “On the Road to Quitting” that has been developed by Health Canada for young adult smokers.	Continuous self-reported abstinence at 6 months; self-reported 30-day PPA^b^ from smoking at 3 and 6 months, operationalized as not having smoked any cigarettes, even a puff, or used other tobacco in the last 30 days; self-reported 7-day PPA at 3 and 6 months.
Bendtsen et al [20], 2021, Sweden, RCT	535	High school students, median 17 (IQR 16-18)	The intervention starts with a 1-week motivational phase. After 1 week the core intervention begins and runs for 12 weeks. Participants receive up to 4 texts per day during the first few weeks, and then the number of messages per day decreases.	Self-help materials: The control group could use the website of the national quit line or contact their high school’s health service for more help	Prolonged abstinence and point prevalence of smoking cessation at the 3- and 6-month follow-ups. Prolonged abstinence, following the Russel standard, was defined at the 3-month follow-up as not smoking more than 5 cigarettes in the past 8 weeks. At 6 months, the definition was altered to not smoking more than 5 cigarettes in the past 5 months. This outcome thus measures abstinence from the start of the 12-week smoking cessation program, allowing for a 4-week grace period. Point prevalence of smoking cessation, a recommended outcome by the Society for Research on Nicotine and Tobacco, was defined as not having smoked a single cigarette in the past 4 weeks. This question measures current behavior and, thus, was the same at both the 3- and 6-month follow-ups.
Chulasai et al [24], 2022, Thailand, RCT	273	18-24	The Quit with US app integrated with smoking cessation counseling from pharmacists	Smoking cessation counseling from pharmacists	The primary smoking abstinence outcome was the 7-day point prevalence at the 12-week follow-up, as recommended for smoking abstinence measures. The outcome was defined as a self-report of the previous 7 consecutive days of continuous abstinence from smoking plus an exhaled CO concentration level of ≤6 ppm.
Crane et al [23], 2018, online, RCT	18,400	18-30	Full version of the smoke-free app	Reduced version that did not include the missions	The primary outcome measure was self-reported continuous abstinence up to the 12-week follow-up.
Graham et al [28], 2021, United States, RCT	2588	18-24	This is Quitting (texting)	Assessment only control	The primary outcome was self-reported 30-day PPA at 7 months analyzed under an intention-to-treat analysis, which counted nonresponders as vaping. Secondary outcomes were 7-day PPA under the intention-to-treat analysis and retention weighted complete case analysis of 30-day and 7-day PPA.
Haug et al [29], 2013, Switzerland, cluster RCT	755	Vocational school students, mean 18.2 (SD 2.3)	Individualized texts to support smoking cessation (2 texts per week for a period of 3 months); possibility to register for a more intensive program providing strategies for smoking cessation around a self-defined quit date (2 texts per day for a period of 4 weeks)	Assessment only control group	7-day point prevalence smoking abstinence at the 6-month follow-up (ie, not having smoked a puff within the past 7 days preceding the follow-up) and 4-week point prevalence smoking abstinence were assessed
Liao et al [22], 2018, China, RCT	344	18-30	Mobile phone–based texts (3-5 messages per day); interventions (Happy Quit) for smoking cessation for the 12-week and 24-week follow-up	No cessation message intervention: Texts every week thanking them for being in the study and texts for assessment	Biochemically verified continuous smoking abstinence at 24 weeks; self-reported 7-day PPA (ie, not even a puff of smoke, for the last 7 days) at 1, 4, 8, 12, 16, 20, and 24 weeks; self-reported continuous abstinence at 4, 12, and 24 weeks.
Mays et al [15], 2021, United States, RCT	349	18-30	6-week mobile messaging intervention	Assessments only	Self-reported cessation was assessed at 6 weeks, 3 months, and 6 months.
Müssener et al [10], 2016, Sweden, RCT	1590	Mainly between 21 and 30	Texts: Those in group 1 received motivational messages (the intervention) 5 times a day for 3 days before their stated quit day and then continue to receive 3-5 motivational texts per day for week 1, 2-4 messages per day for the next 2-4 weeks, and then 10 messages per week for the remaining 8 weeks.	Texts unrelated to quitting	At the 4-month follow-up, 8 weeks of prolonged abstinence (having smoked ≤5 cigarettes during this time); self-reported 4-week PPA (not having smoked a single cigarette); self-reported 7-day PPA (defined as not smoking any cigarettes in the past 7 days)
Palmer et al [31], 2022, United States, RCT	27	17-21	Contingency management was delivered through DynamiCare Health’s smartphone app for 4 weeks, in which financial incentives were delivered contingent on abstinent cotinine samples after the quit day until the end of treatment.	Assessment only: Control participants earned incentives for submitting cotinine, regardless of abstinence.	Abstinence at 1-month follow-up
Shi et al [32], 2013, China, cluster RCT	179	16-19	Tailored information via mobile phone texts for 12 weeks	A self-help pamphlet about smoking cessation	Self-reported 7- and 30-day abstinence at 12-week follow-up
Villanti et al [33], 2022, United States, RCT	437	18-30	12-week tailored text smoking-cessation program with a companion web-based intervention	Referral to online quit resources	Self-reported 30- and 7-day PPA at 12-week follow-up
Ybarra et al [34], 2013, United States, RCT	164	18-25	SMS USA, a 6-week smoking cessation intervention. Intervention participants received texts daily pre- and postquitting. Everyone receives messages 14 days prior to the quit day and through the day after the quit day. Then, participants are ”pathed“ to particular messages based upon their self-reported smoking status on day 2 and day 7 post quit. Those who are successful at quitting receive messages aimed at relapse prevention, whereas those who have slipped receive messages aimed at getting the person to recommit to quitting and trying again. Other name: Stop My Smoking USA	No intervention: Attention-matched control; messages aimed at improving one's sleep and increasing one's fitness, along with general messages about the most well-known health dangers of smoking. Messages sent on the same schedule as the intervention group.	Three-month continuous abstinence; smoking ≤5 cigarettes since the quit day 4 weeks post quit; 7-day PPA at 4 weeks

a RCT: randomized controlled trial.

b PPA: point prevalence abstinence.

Most studies [10,15,19,22,24,31,33,34] provided incentives to participants in the form of financial rewards for follow-up, with the highest incentive being US $310 [31]. One study offered an alternative incentive in the form of a lottery draw at the end of the study instead of a monetary incentive. Nine of the selected studies provided a tailored intervention to participants [15,19,22,24,28,29,32-34]. These interventions were tailored to the user’s age, stage of quitting, smoking history, stage of readiness to quit, demographics, etc.

Nine studies used SMS text messaging–based interventions [10,15,20,22,28,29,32-34], and 4 studies used app-based interventions [19,23,24,31]. The duration of the smoking cessation intervention varied from 5 days to 6 months, with 8 studies lasting ≥12 weeks [10,19,20,22,24,29,32,33], and the remaining studies lasting between 5 days and 6 weeks [15,23,28,31,34]. Most studies used an inactive control, such as an assessment-only control group [15,28,29,31], SMS text messages that were unrelated to quitting [10,22,34], or self-help cessation materials [19,20,32,33]. One study [23] provided a reduced version app that did not include the mission. One study [24] provided the control with smoking cessation counseling from a pharmacist. The majority of studies used self-reported abstinence, without biochemical verification. Four studies [22,24,31,33] used biochemically verified abstinence, such as salivary cotinine test [31,33], urine cotinine test [22], and exhaled CO concentration for verification [24].

### Risk of Bias

**Figure 2.**
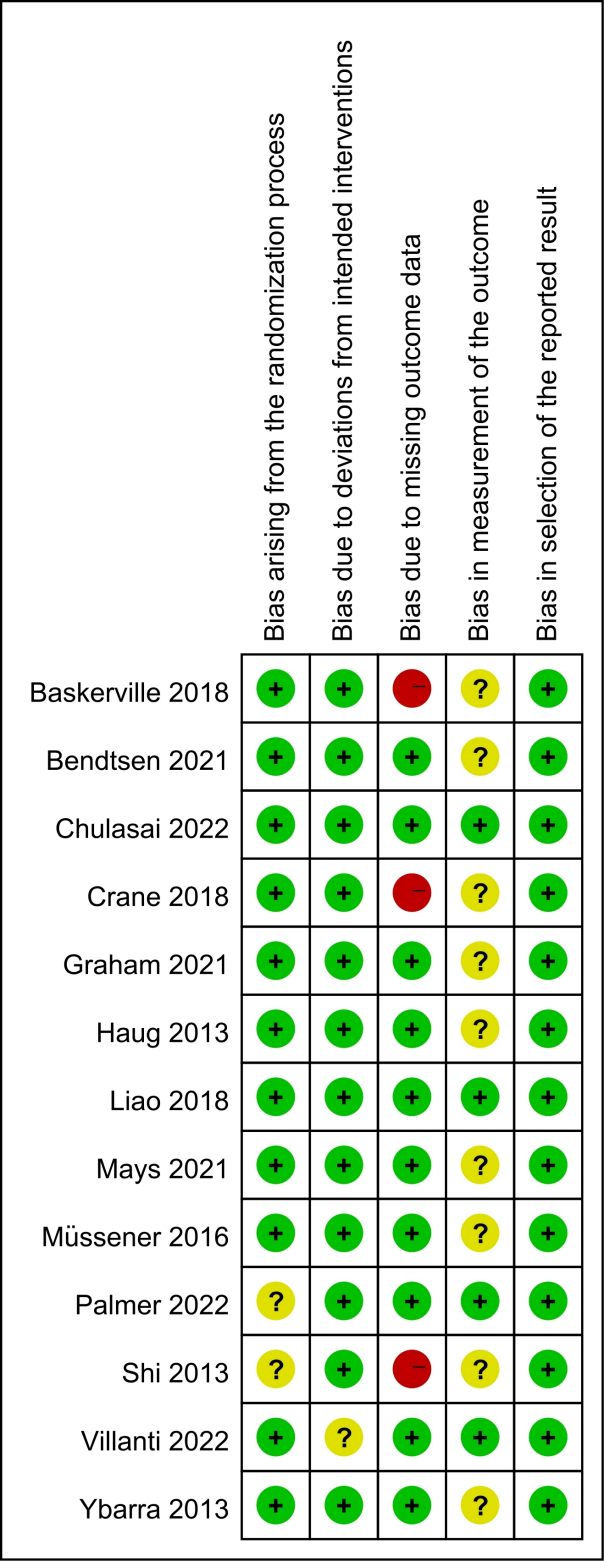
Risk of bias summary: reviewers’ judgments about each risk of bias item for each included study [10,15,19,20,22-24,28,29,31-34].

The risk of bias assessments for individual studies is shown in Figure 2. The majority of studies reported methods of randomization and allocation concealment that were judged to be of low risk for the randomization process. The main source of some concerns was the measurement of the outcome, as these studies used self-reported smoking cessation rates without biochemical validation, and the intervention could not be blinded to participants due to its inherent characteristics. We judged 3 studies [19,23,32] to be at a high risk of bias for missing outcome data because more than 50% of participants were lost to follow-up or the difference in follow-up rates between study arms was more than 20%. The remaining studies were judged to be at a low risk in the domain of missing outcome data. Overall, 2 studies were at a low risk of bias (judged at low risk for all domains) [22,24], 3 were at high risk (judged to be at high risk in at least one domain) [19,23,32], and the remaining studies were of some concern.

### Continuous Abstinence

**Figure 3.**
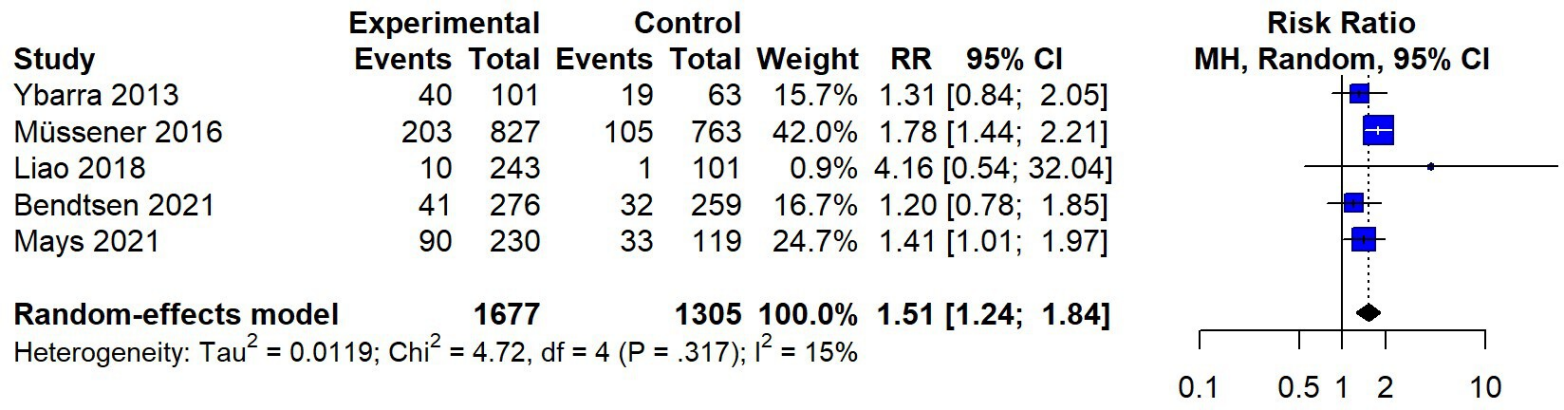
Random-effects meta-analysis for SMS text messaging compared to an inactive control on continuous abstinence [10,15,20,22,34]. MH: Mantel-Haenszel; RR: risk ratio.

**Figure 4.**
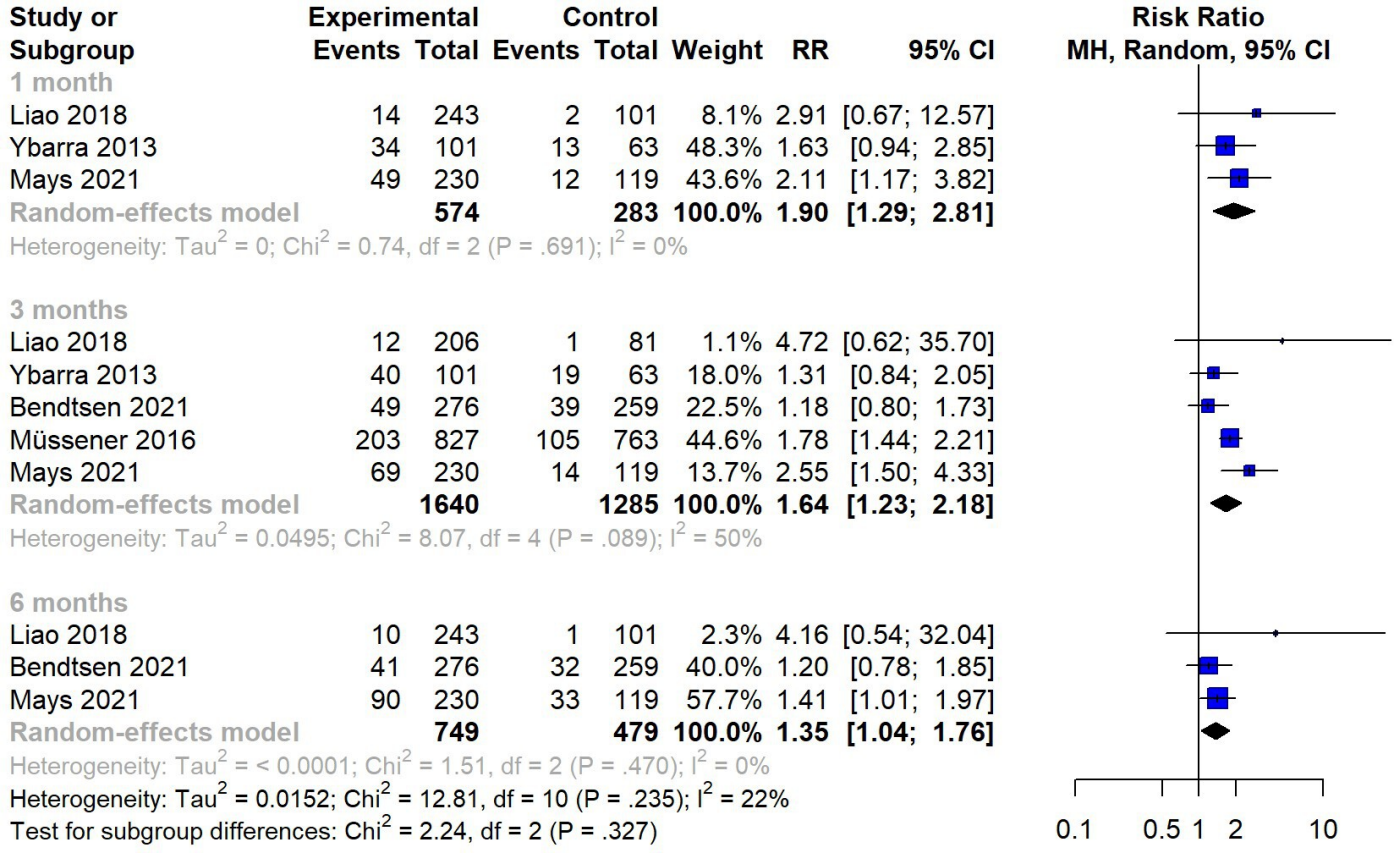
Subgroup analysis: random-effects meta-analysis for SMS text messaging compared to an inactive control on continuous abstinence at the 1-, 3-, and 6-month follow-ups [10,15,20,22,34]. MH: Mantel-Haenszel; RR: risk ratio.

The result of continuous abstinence is illustrated in Figure 3. Combining data from 5 studies using a random-effects meta-analysis, a significant improvement in continuous abstinence rates was observed with SMS text messaging interventions, with an RR of 1.51 (95% CI 1.24-1.84) compared with inactive control conditions (assessment only, non–quit-related SMS text messages, or self-help materials). For continuous abstinence, no high-risk study was identified when comparing the SMS text message intervention with the inactive control. The subgroup analysis (Figure 4) showed a pooled RR of 1.90 (95% CI 1.29-2.81) for continuous abstinence at the 1-month follow-up, with no significant heterogeneity observed among the included studies. At the 3-month follow-up, the pooled RR for SMS text messaging interventions versus an inactive control was 1.64 (95% CI 1.23-2.18), with an *I*^2^ value of 50.4% (*P*=.089). At the 6-month follow-up, the SMS text messaging intervention yielded similar results as the 3-month follow-up for continuous abstinence (RR 1.35, 95% CI 1.04-1.76), with no significant heterogeneity observed (*I*^2^=0.0%).

**Figure 5.**
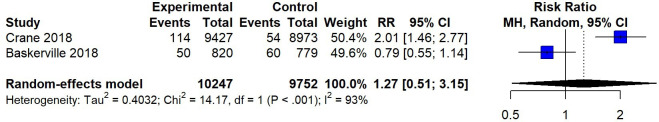
Random-effects meta-analysis for apps compared to a control on continuous abstinence [19,23]. MH: Mantel-Haenszel; RR: risk ratio.

Only 2 studies provided data on the RR of continuous abstinence in the comparison between app-based interventions and controls (Figure 5). However, these studies yielded conflicting results. It is worth noting that both studies included in this analysis were subject to a high risk of bias due to missing outcome data.

### 7-Day PPA

**Figure 6.**
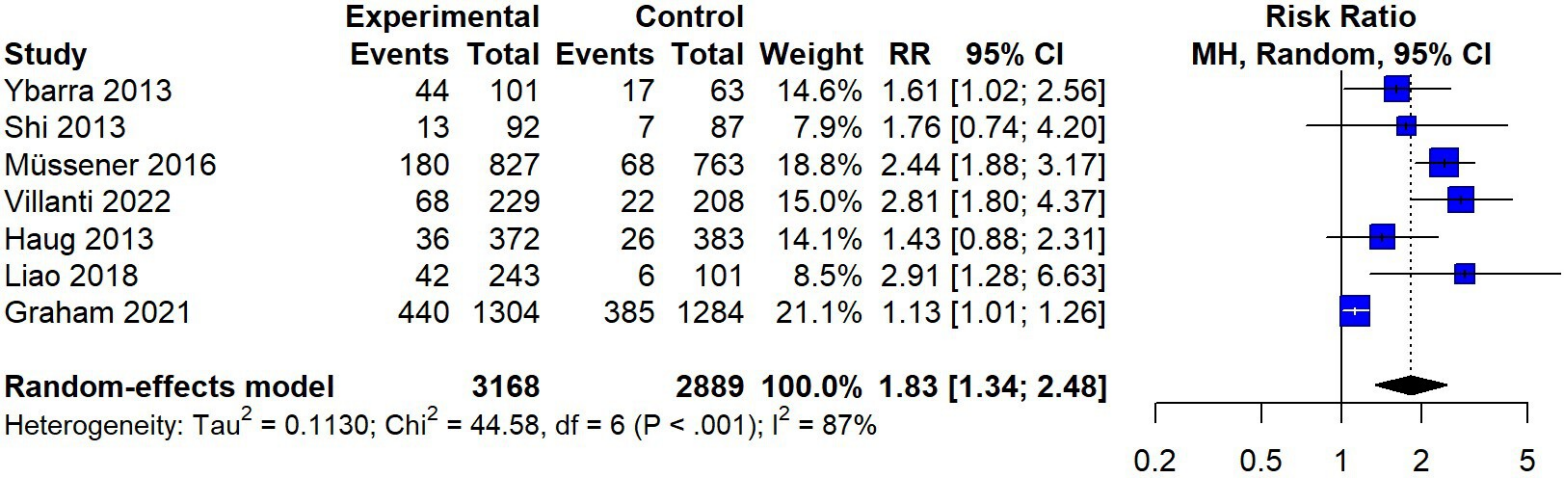
Random-effects meta-analysis for SMS text messaging compared to an inactive control on 7-day point prevalence abstinence [10,22,28,29,32-34]. MH: Mantel-Haenszel; RR: risk ratio.

**Figure 7.**
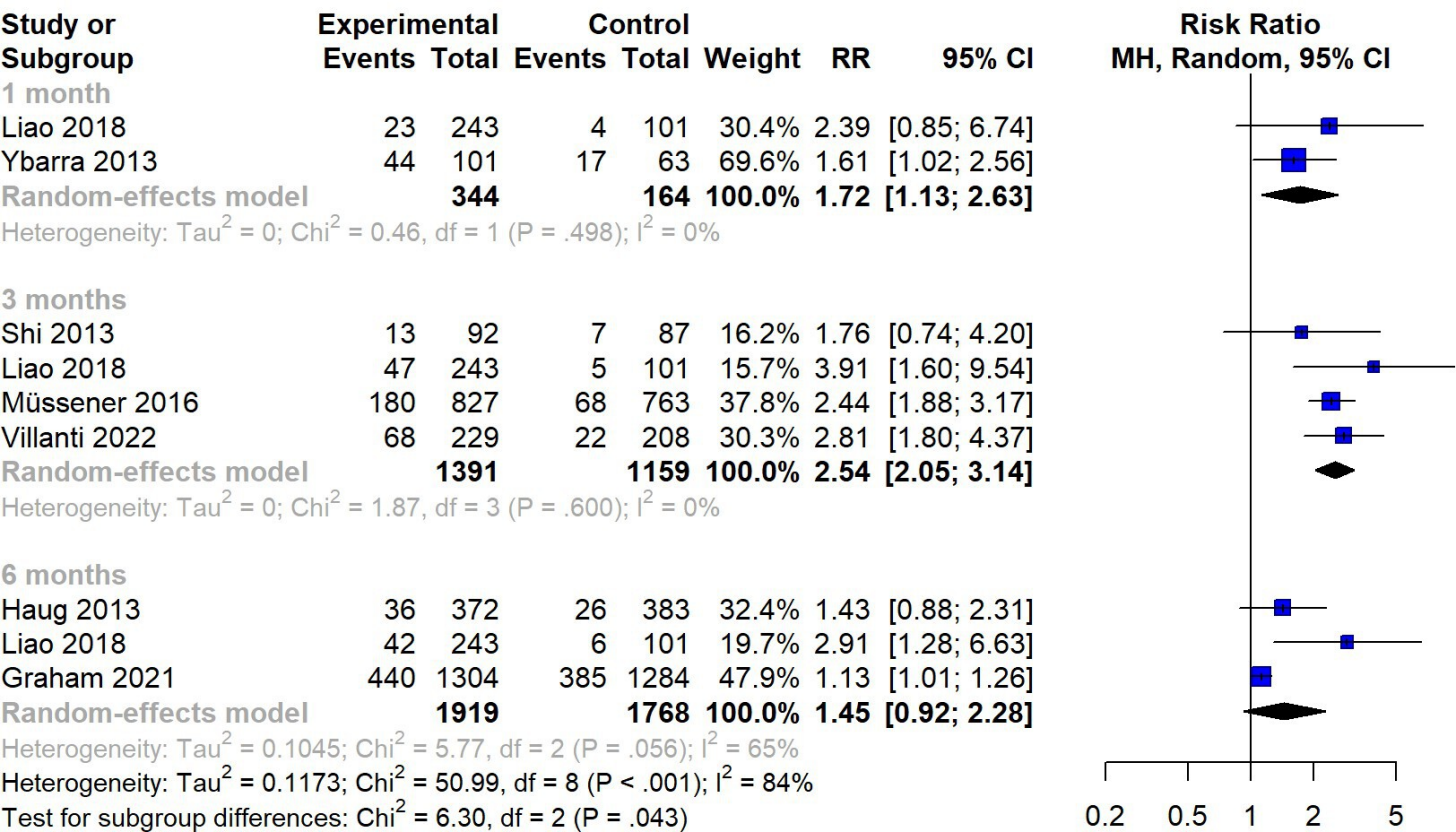
Subgroup analysis: random-effects meta-analysis for SMS text messaging compared to an inactive control on the 7-day point prevalence abstinence at the 1-, 3-, and 6-month follow-ups [10,22,28,29,32-34]. MH: Mantel-Haenszel; RR: risk ratio.

In terms of 7-day PPA, as illustrated in Figure 6, the meta-analysis of 7 studies showed an RR of 1.83 (95% CI 1.34-2.48), with an *I*^2^ value of 87% (*P*<.001). A sensitivity analysis excluding high-risk studies showed consistent results (RR 1.84, 95% CI 1.32-2.57; *k*=6), indicating the stability of the results. The subgroup analysis (Figure 7) showed that SMS text messaging interventions had a significant impact at the 1- and 3-month follow-ups, with pooled RRs of 1.72 (95% CI 1.13-2.63) and 2.54 (95% CI 2.05-3.14), respectively, compared with inactive control conditions. When pooling across 3 studies, SMS text messaging interventions showed nonsignificant efficacy in promoting 7-day PPA at the 6-month follow-up (RR 1.45, 95% CI 0.92-2.28).

**Figure 8.**
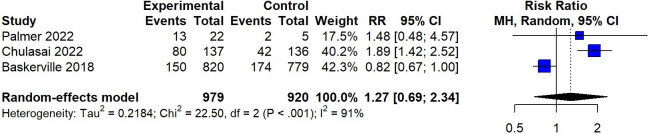
Random-effects meta-analysis for apps compared to a control on 7-day point prevalence abstinence [19,24,31]. MH: Mantel-Haenszel; RR: risk ratio.

Pooling across 3 studies, app-based interventions showed no significant efficacy in promoting 7-day PPA (RR 1.27, 95% CI 0.69-2.34), indicating a lack of substantial impact (Figure 8). Notably, a high level of heterogeneity was observed among the included studies, with an *I*^2^ value of 91% (*P*<.001), suggesting significant variation in the results. A sensitivity analysis was conducted by removing the study identified as having a high risk of bias. After the exclusion, the remaining studies showed a pooled RR of 1.86 (95% CI 1.41-2.46), indicating a relatively higher effect size in favor of app-based interventions. Importantly, the removal of the high-risk study resulted in a substantial decrease in heterogeneity, from 91% to 0%.

## Discussion

### Principal Results

This study aimed to synthesize the published literature on the efficacy of mobile phone–based interventions for smoking cessation among young people. Our findings suggest that SMS text messaging interventions could be effective for smoking cessation among young individuals, whereas the evidence for app-based interventions is inconclusive. The sensitivity analysis showed stable results for SMS text messaging interventions, but conflicting results for the app-based study.

### Comparison With Prior Work

Previous reviews of mobile phone–based interventions have varied considerably in terms of population characteristics [35-39] and geographical limitations [40,41]. To our knowledge, there is currently no meta-analysis supporting the efficacy of mobile phone–based interventions among young people. Our review fills this gap by providing evidence that SMS text messaging approaches to smoking cessation are robust among young people. Due to the nontemporal and nonspatial nature of mobile phone–based interventions, they can reach a wider audience and serve as a good adjunct to smoking cessation interventions for this population.

The meta-analysis showed that SMS text messaging smoking cessation interventions were effective for continuous abstinence among young people, which is in line with a previous study on general smokers [18]. The RR value at 6 months of continuous abstinence was slightly lower than that for general smokers (RR 1.54, 95% CI 1.19-2.00) [18]. This discrepancy may be attributed to the interventions lacking specific tailoring to address the unique characteristics and needs of young individuals. Smoking cessation was related to motivation to quit, which can differ across age groups [42]. A nationwide study conducted in the United States revealed the common reasons for smoking cessation in young adults aged 18-34 years; the two most popular reasons were physical fitness (64%) and the cost of tobacco (64%). More than half of current smokers also identified “encouragement from friend or relative” (55.2%) and “info about health hazards” (59.7%) as reasons for quitting smoking [43]. Despite most of the included studies in our analysis allowing for customization of the quit date, the interventions’ content may not have been specifically tailored for young people. Developing more targeted cessation interventions that take into account young people’s unique motivations and use patterns is crucial. This approach may help promote a positive attitude toward quitting smoking [44]. Furthermore, the efficacy of smoking cessation interventions among young people was observed to be slightly higher at 3 months compared to 1 month, as indicated by the 7-day PPA. This finding aligns with the cycle of withdrawal response, with the most intense withdrawal response occurring in the weeks when smokers first attempt to quit [45].

Less than one-third of smokers use cessation medications or behavioral counseling to support quit attempts [46]. Young smokers are more reluctant to seek treatment for smoking cessation than older smokers [47]. Pharmacotherapy and counseling often require face-to-face contact and the presence of a health care provider, which can be time-consuming and may not be covered by insurance. Additionally, adverse drug reactions to medication can be a barrier to quitting [48]. Young people are more likely to use mobile phones and novel technology in their daily lives [16]. Mobile phone–based interventions can be delivered remotely and offer a more cost-effective, discreet, convenient, and accessible alternative. Therefore, mobile phone–based smoking cessation interventions are a promising alternative for young people.

In recent years, the development of technology has led to the increasing popularity of mobile phone apps designed for health management [49,50]. The included studies were published between 2012-2022 for the SMS text messaging–based studies and between 2018-2022 for the app-based studies, indicating a relatively recent focus on app-based interventions. Mobile phone apps can provide more interactive and personalized features than SMS text messaging, such as tracking progress, setting goals, and sending notifications [23]. Apps can also offer real-time support and a wealth of resources, including educational materials, coping strategies, and social support networks [24]. However, the existing literature on app-based interventions exhibits significant heterogeneity and conflicting results. The lack of standardized protocols and guidelines for app-based smoking cessation interventions further exacerbates the challenge of generating consistent evidence. Therefore, there is a need for further research on smoking cessation apps targeted toward young people. In particular, future studies should explore the optimal features and design of smoking cessation apps.

Despite its many advantages, high dropout [51] and nonadherence [52] remain significant limitations. Incentives were offered in more than half of the included trials, and these studies generally had lower dropout rates than those without incentives. The majority of included studies with incentives had loss to follow-up rates ranging from 6% to 24%, while studies without incentives had rates between 26% and 92%. We note that the study by Crane et al [23], which was conducted in a real-world setting, reported the highest rate of missing data. This finding was in line with previous research highlighting the challenge of retaining participants in real-world studies [53]. A review showed that the personalization of content, app design, reminder form, and personal support help to improve adherence, but research on factors that influence adherence to mobile health apps remains limited [52]. Increasing people’s engagement and retention is important as a key consideration for such interventions on a large scale in the real world. Future research should focus on identifying the most effective design, personalized content, features, and support mechanisms to increase their uptake and intention.

### Limitations

Several limitations must be considered when interpreting the findings of this review. First, few studies examined app-based interventions, which limited the amount of available evidence for analysis and interpretation. Furthermore, considerable heterogeneity was observed among app-based studies. This scarcity of data highlights the need for further research in this area to improve the understanding of the efficacy of app-based interventions for smoking cessation. Second, the studies included in this review were largely conducted in high-income countries, limiting the generalizability of the findings to other settings. Lastly, a significant number of studies relied on self-reported abstinence without biochemical validation, raising some concerns in the domain of outcome measurement. In light of these limitations, future research should aim to address these issues and ensure the production of high-quality evidence to guide mobile phone–based smoking cessation interventions.

### Conclusions

Our meta-analysis provides evidence that SMS text messaging smoking cessation interventions are effective among young people. There is a need for further research on smoking cessation apps, especially those targeted at young people. Future research should also focus on identifying the most effective mobile phone–based cessation approaches and on developing strategies to increase their uptake and intention.

## Supplementary material

10.2196/48253 Multimedia Appendix 1 Search strategies of PubMed, Web of Science, Embase, and the Cochrane Library.

10.2196/48253 Checklist 1 PRISMA (Preferred Reporting Items for Systematic Reviews and Meta-Analyses) checklist

## Abbreviations

MH: Mantel-Haenszel
PPA: point prevalence abstinence
PRISMA: Preferred Reporting Items for Systematic Reviews and Meta-Analyses
RCT: randomized controlled trial
RR: risk ratio
