# Supplementary material for: Mobile Phone–Based Interventions for Smoking Cessation Among Young People: Systematic Review and Meta-Analysis
Source: JMIR Mhealth Uhealth. 2023 Sep 12;11:e48253. doi: 10.2196/48253 (PMC10510452; doi:10.2196/48253)
Supplement: Multimedia Appendix 1 [file mhealth-v11-e48253-s001.docx]

**Multimedia Appendix 1**

Searching strategies in Pubmed n=171

((smoking cessation[MeSH Terms]) AND (((young) OR (student)) OR (adolescent))) AND ((((text messaging[Title/Abstract]) OR (phone-based[Title/Abstract]) OR (smartphone[Title/Abstract]) OR (app[Title/Abstract]) OR (mobile health[Title/Abstract]) OR sms[Title/Abstract] OR txt[Title/Abstract])))

Searching strategies in Cochrane n=147

Trials matching "#3 - ((text messaging) OR (phone-based) OR (smartphone) OR (app) OR (mobile health) OR sms OR txt):ti,ab,kw AND (((young) OR (student)) OR (adolescent)) AND (smoking cessation):ti,ab,kw" in Trials with 'Tobacco Addiction' in Cochrane Groups (Word variations have been searched)

Searching strategies in Web of Science n = 398

((TS=((((text messaging) OR (phone-based) OR (smartphone) OR (app) OR (mobile health) OR sms OR txt)))) AND TS=((((young) OR (student)) OR (adolescent)))) AND TS=((smoking cessation)) and Preprint Citation Index (Exclude – Database)

Searching strategies in Embase n = 330

#1 'smoking cessation'/exp OR 'smoking cessation' OR (('smoking'/exp OR smoking) AND ('cessation'/exp OR cessation))

#2 text AND messaging OR 'phone based' OR smartphone OR app OR (mobile AND health) OR sms OR txt

#3 young OR student OR adolescent

#4 #1 AND #2 AND #3
